# Traumatic brain injury—the effects of patient age on treatment intensity and mortality

**DOI:** 10.1186/s12883-020-01943-6

**Published:** 2020-10-17

**Authors:** Ola Skaansar, Cathrine Tverdal, Pål Andre Rønning, Karoline Skogen, Tor Brommeland, Olav Røise, Mads Aarhus, Nada Andelic, Eirik Helseth

**Affiliations:** 1 Institute of Clinical Medicine, University of Oslo, Oslo, Norway; 2 Department of Neurosurgery, Oslo University Hospital, Oslo, Norway; 3 Department of Radiology and Nuclear Medicine, Oslo University Hospital, Oslo, Norway; 4 Division of Orthopaedic Surgery, Oslo University Hospital, Oslo, Norway; 5 Department of Physical Medicine and Rehabilitation, Oslo University Hospital, Oslo, Norway; 6 Research Centre for Habilitation and Rehabilitation Models and Services (CHARM), Faculty of Medicine, Institute of Health and Society, University of Oslo, Oslo, Norway

**Keywords:** Traumatic brain injury, Neurosurgery, Age, Treatment, Mortality, Management intensity

## Abstract

**Background:**

Ageing is associated with worse treatment outcome after traumatic brain injury (TBI). This association may lead to a self-fulfilling prophecy that affects treatment efficacy. The aim of the current study was to evaluate the role of treatment bias in patient outcomes by studying the intensity of diagnostic procedures, treatment, and overall 30-day mortality in different age groups of patients with TBI.

**Methods:**

Included in this study was consecutively admitted patients with TBI, aged ≥ 15 years, with a cerebral CT showing intracranial signs of trauma, during the time-period between 2015–2018. Data were extracted from our prospective quality control registry for admitted TBI patients. As a measure of management intensity in different age groups, we made a composite score, where placement of intracranial pressure monitor, ventilator treatment, and evacuation of intracranial mass lesion each gave one point. Uni- and multivariate survival analyses were performed using logistic multinomial regression.

**Results:**

A total of 1,571 patients with TBI fulfilled the inclusion criteria. The median age was 58 years (range 15–98), 70% were men, and 39% were ≥ 65 years. Head injury severity was mild in 706 patients (45%), moderate in 437 (28%), and severe in 428 (27%). Increasing age was associated with less management intensity, as measured using the composite score, irrespective of head injury severity. Multivariate analyses showed that the following parameters had a significant association with an increased risk of death within 30 days of trauma: increasing age, severe comorbidities, severe TBI, Rotterdam CT-score ≥ 3, and low management intensity.

**Conclusion:**

The present study indicates that the management intensity of hospitalised patients with TBI decreased with advanced age and that low management intensity was associated with an increased risk of 30-day mortality. This suggests that the high mortality among elderly TBI patients may have an element of treatment bias and could in the future be limited with a more aggressive management regime.

## Background

Traumatic brain injury (TBI) is a serious public health and societal problem and a major cause of injury-related deaths and disability [1–3]. In the last decades, the typical patient with TBI has changed from a young male, injured in a high-energy trauma, to an elderly man or woman, often with significant comorbidity, injured in a low-energy fall [2–4]. Elderly patients with TBI tend to have a higher initial Glasgow Coma Scale score (GCS) than young patients, and they are less frequently multi-traumatised [2, 3, 5, 6]. Nevertheless, older age is associated with worse treatment outcome after TBI [7–10]. This may be a direct consequence of biological aging and pre-injury comorbidities [11–14]. However, the association between greater age and poor outcome could also lead to the assumption of futility with regard to immediate thorough diagnostic work-up, neurosurgical procedures, neurointensive treatment, and rehabilitation [15]. Treatment limiting decisions (TLDs) are more often made for older patients with TBI than for the young [16, 17]. Thus, for some older patients with TBI, limited diagnostic work-up, early TLDs (before 48 h), and less rehabilitation may reduce their possibility for survival and recovery. Over time, the high mortality and morbidity among older patients with TBI may start to function as a self-fulfilling prophecy (i.e., a sociological term used to describe a belief that influences people's behaviour in such a way as to align with that belief and fulfil it). In light of recent data indicating the benefit of aggressive treatment and rehabilitation in older patients with TBI [18–23], we evaluated the possibility of a self-fulfilling prophecy negatively impacting current TBI treatment regimes and outcomes among older patients.

In this registry study of hospital-admitted TBI patients ≥ 15 years of age we investigated the potential association between management intensity and risk of 30-day mortality in different age groups.

## Methods

### Study type

This is a retrospective study of 1,571 consecutive patients with TBI, aged ≥ 15 years, treated at the Department of Neurosurgery in Oslo University Hospital (OUH) as inpatients in the acute phase, in a four year time period between 2015–2018.

### Study setting

OUH is the only Level I trauma centre and the only hospital with neurosurgical service in the South-East region of Norway, which had a population of 3.0 million in 2018 [24]. There are 19 local hospitals in this region that are involved in primary care of trauma patients. Trauma patients with severe injuries and patients with suspected severe TBI are admitted directly to OUH. Patients with less severe injuries are admitted to local hospitals but transferred to OUH if in need of neurosurgical surveillance and/or neurosurgical procedures. OUH also serves as the primary trauma referral hospital for Oslo, the population of which was 673,469 in 2018 [24]. Treatment of TBI at OUH follows guidelines made by the Brain Trauma Foundation and the Scandinavian Neurotrauma Committee [25, 26].

### Study database

The Oslo TBI Registry—Neurosurgery is a prospective quality control database run by the neurosurgical department at OUH since January 1, 2015. Data is derived from electronic medical records and stored in the MedInsight database platform. The registry has been approved by an OUH data protection officer (DPO approval number 2016/17,569). Included in the registry are patients fulfilling the following four criteria: (a) TBI, (b) cerebral-CT/cerebral-CT-Angiography or cerebral-MRI/cerebral-MR-Angiography showing signs of acute trauma (haemorrhage, fracture, traumatic axonal injury, vascular injury), (c) admitted to OUH as an inpatient within seven days after injury, and (d) having a Norwegian social security number.

### Independent variables

The following data was extracted: date of injury, injury mechanism (fall, motor vehicle, pedestrian, bicycle, sport, violence, self-harm, other), sex, age at time of injury, pre-injury need for assistance in daily life (living at home without assistance, living at home with assistance or being institutionalised), pre-injury American Society of Anaesthesiologists score (ASA) [27], multiple trauma (trauma in two or more body regions—yes/no), Glasgow Coma Scale score (GCS—recorded as lowest score documented in a time point between injury and OUH emergency room or intubation) [28], Head Injury Severity Scale (HISS—mild, moderate or severe) [29], pathoanatomic injury description on primary cerebral-CT, Rotterdam CT-score on primary cerebral-CT [30], trauma team activation (TTA) at admittance to OUH, advanced TBI imaging (cerebral-MR, cerebral-CT-Angiography/cerebral-CT-Venography, or cerebral-MR-Angiography/cerebral-MR-Venography), neurosurgical procedures (invasive intracranial pressure monitoring (ICP), evacuation of intracranial mass lesion, and decompressive craniectomy), ventilator treatment (no/yes), and 30-day mortality.

### The dependent variable

As a measure of management intensity in different age groups, we made a composite score, where placement of ICP-monitor, ventilator treatment, and evacuation of intracranial mass lesion each gave one point. Thus, the score for treatment intensity ranged from 0 to 3.

### Ethics

This study was approved by the OUH DPO (approval number 2017/3904). The study has been presented to the Regional Ethical Committee (REC). REC categorised the study as a Quality Control Study and determined that DPO approval was sufficient.

### Statistics

Descriptive statistics summarise the characteristics of patients, injuries, and treatment. Categorical data are presented as frequencies and percentages. Continuous variables are presented using mean or median, depending on the distribution. To compare group differences, we used the Pearson χ2 test for categorical variables and independent t-test or Mann–Whitney U-Test for continuous variables. The effect of age on the ordinal variable of treatment intensity was investigated using multivariate ordinal regression. The proportional odds assumption was not fulfilled; instead, a multinomial regression was fitted. To ease the interpretation of the model (available upon request), the calculated probabilities from the model are displayed in a graph. A stratified density plot was created in order to verify that the multinomial model is in line with the observed probabilities. Uni- and multivariate logistic regression was used to investigate the effect on 30-day mortality. An overall survival (OS) analysis was conducted using Kaplan–Meier curves, measuring survival from time of injury to time of death.

R v3.6 and STATA SE were used for all analyses. *P*-values < 0.05 were considered significant.

## Results

### Patients

Included in this study were 1,571 consecutive adult patients with TBI (aged ≥ 15 years) and with a cerebral CT showing signs of trauma. Patient characteristics are presented in Table 1. Seventy per cent of the patients were men. The male preponderance was clear in patients aged 15–74 years, while among patients aged 75 years or older there was a gradual shift to a female preponderance. The median age was 58 years (range 15–98). The fractions of patients aged 65 years or older, 75 years or older, and 85 years or older were 39%, 20%, and 8%, respectively.

**Table 1:** Baseline patient characteristics for 1,571 patients by age groups

	**15-24yrs**	**25-34yrs**	**35-44yrs**	**45-54yrs**	**55-64yrs**	**65-74yrs**	**75-84yrs**	** ≥ 85yrs**
**Count (n, %)**	179 (100)	168 (100)	153 (100)	197 (100)	258 (100)	297 (100)	197 (100)	122 (100)
**Male sex (n, %)**	134 (74.9)	134 (79.8)	125 (81.7)	145 (73.6)	195 (75.6)	201 (67.7)	112 (56.9)	46 (37.7)
**Pre-injury ASA-score (n, %)**								
1	165 (92.2)	134 (79.8)	102 (66.7)	114 (57.9)	72 (27.9)	66 (22.2)	18 (9.1)	4 (3.3)
2	11 (6.1)	26 (15.5)	31 (20.3)	45 (22.8)	88 (34.1)	127 (42.8)	86 (43.7)	30 (24.6)
3	3 (1.7)	7 (4.2)	20 (13.1)	38 (19.3)	97 (37.6)	97 (32.7)	91 (46.2)	85 (69.7)
4	0 (0)	1 (0.6)	0 (0)	0 (0)	1 (0.4)	7 (2.4)	2 (1.0)	3 (2.5)
**Pre-injury living (n, %)**								
Independent	176 (98.3)	162 (96.4)	147 (96.1)	185 (93.9)	236 (91.5)	269 (90.6)	155 (78.7)	51 (41.8)
Home with assistance	1 (0.6)	5 (3.0)	4 (2.6)	8 (4.1)	20 (7.8)	22 (7.4)	29 (14.7)	54 (44.3)
Institutionalised	2 (1.1)	1 (0.6)	2 (1.3)	4 (2.0)	2 (0.8)	6 (2.0)	13 (6.6)	17 (13.9)
**Pre-injury antithrombotics (n, %)**								
Anticoagulation	1 (0.6)	0 (0)	5 (3.3)	2 (1.0)	14 (5.4)	33 (11.1)	52 (26.4)	38 (31.1)
Platelet inhibitor	0 (0)	0 (0)	1 (0.7)	10 (5.1)	38 (14.7)	87 (29.3)	68 (34.5)	45 (36.9)
Platelet inhibitor + anticoagulation	0 (0)	0 (0)	1 (0.7)	1 (0.5)	3 (1.2)	7 (2.4)	7 (3.6)	2 (1.6)
**GCS (mean)**	10.0	11.0	11.1	11.0	11.2	11.8	11.4	12.5
**HISS (n, %)**								
Mild	62 (34.6)	83 (49.4)	68 (44.4)	81 (41.1)	111 (43.0)	134 (45.1)	94 (47.7)	73 (59.8)
Moderate	50 (27.9)	32 (19.0)	41 (26.8)	58 (29.4)	74 (28.7)	100 (33.7)	50 (25.4)	32 (26.2)
Severe	67 (37.4)	53 (31.5)	44 (28.8)	58 (29.4)	73 (28.3)	63 (21.2)	53 (26.9)	17 (13.9)
**Pathoanatomy (n, %)**								
Skull fracture	121 (67.6)	107 (63.7)	95 (62.1)	125 (63.5)	135 (52.3)	133 (44.8)	56 (28.4)	36 (29.5)
SDH	72 (40.2)	73 (43.5)	65 (42.5)	109 (55.3)	169 (65.5)	192 (64.6)	133 (67.5)	77 (63.1)
tSAH	80 (44.7)	104 (61.9)	93 (60.8)	116 (58.9)	170 (65.9)	189 (63.6)	118 (59.9)	72 (59.0)
EDH	47 (26.3)	35 (20.8)	34 (22.2)	36 (18.3)	33 (12.8)	28 (9.4)	9 (4.6)	5 (4.1)
Brain contusion	100 (55.8)	82 (48.8)	82 (53.6)	102 (51.8)	138 (53.5)	137 (46.1)	87 (44.2)	48 (39.3)
**Rotterdam CT score (n, %)**								
1,2	103 (57.5)	82 (48.8)	60 (39.2)	76 (38.6)	83 (32.2)	89 (30.0)	44 (22.3)	38 (31.1)
3,4	66 (36.9)	67 (39.9)	80 (52.3)	101 (51.3)	152 (58.9)	182 (61.3)	132 (67.0)	73 (59.8)
5,6	10 (5.6)	19 (11.3)	13 (8.5)	20 (10.2)	23 (8.9)	26 (8.8)	21 (10.7)	11 (9.0)
**Multiple trauma (n, %)**								
Yes	101 (56.4)	94 (56.0)	78 (51.0)	122 (61.9)	134 (51.9)	127 (42.8)	76 (38.6)	40 (32.8)
No	78 (43.6)	74 (44.0)	75 (49.0)	75 (38.1)	124 (48.1)	170 (57.2)	121 (61.4)	82 (67.2)
**Management intensity (n, %)**								
TTA	165 (92.2)	140 (83.3)	129 (84.3)	167 (84.8)	214 (82.9)	205 (69.0)	127 (64.5)	69 (56.6)
CTA/CTV/MRA/MRV	112 (62.6)	97 (57.7)	84 (54.9)	123 (62.4)	164 (63.6)	163 (54.9)	84 (42.6)	21 (17.2)
MR	90 (50.3)	52 (31.0)	52 (34.0)	69 (35.0)	75 (29.1)	50 (16.8)	21 (10.7)	4 (3.3)
Ventilator therapy	97 (54.2)	69 (41.1)	65 (42.5)	89 (45.2)	119 (46.1)	112 (37.7)	69 (35.0)	22 (18.0)
ICP-monitor	62 (34.6)	40 (23.8)	42 (27.5)	59 (29.9)	81 (31.4)	59 (19.9)	27 (13.7)	3 (2.5)
Evacuation of mass lesion	18 (10.1)	22 (13.1)	27 (17.6)	27 (13.7)	43 (16.7)	49 (16.5)	20 (10.2)	10 (8.2)
Decompressive craniectomy	4 (2.2)	4 (2.4)	6 (3.9)	9 (4.6)	4 (1.6)	4 (1.3)	0 (0)	0 (0)

*ASA* American Society of Anesthesiologists, *LOC* Loss of consciousness, *GCS* Glasgow Coma Scale, *HISS* Head Injury Severity Scale, *SDH* Subdural haematoma, *tSAH* Traumatic subarachnoidal haemorrhage, *EDH* Epidural haematoma, *TTA* Trauma team activation, *CTA* Computed tomography angiography, *CTV* Computed tomography venography, *MRA* Magnetic resonance angiography, *MRV* Magnetic resonance venography, *MR* Magnetic resonance, *ICP* Intracranial pressure monitor

**Fig. 1:**
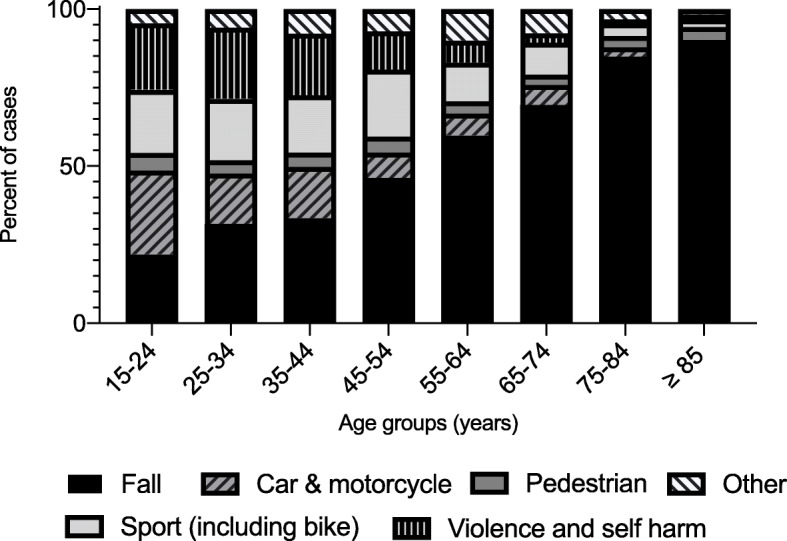
Injury mechanism in different age groups

Increasing age was significantly associated with a higher comorbidity (ASA score 3–4) (*p* < 0.001), need for assistance in daily life (*p* < 0.001), and the use of antithrombotic medication (*p* < 0.001). Falls were the most frequent injury mechanism (55%), followed by motor vehicle accidents (10%), sports accidents (including bicycles) (9%), and violence/self-harm (10%). The proportion of fall-related injuries increased significantly with increasing age (*p* < 0.001) (Fig. 1).

### Head injury severity

Mean GCS score, HISS severity grade, and Rotterdam CT-score for the different age groups are presented in Table 1. The mean GCS score and the fraction of mild TBI increased with increasing age (*p* < 0.03), indicating less severe TBI with increasing age. In contrast, the Rotterdam CT-score showed a gradual increase with increasing age, indicating more severe injury in the higher age groups (*p* < 0.001). The fraction of patients with multiple trauma decreased with increasing age (*p* < 0.001).

### Patient management

The trauma team was activated for initial triage in 77% of the patients, advanced TBI imaging was performed in 60%, ICP-monitoring was used in 24%, ventilator treatment was applied to 41%, surgical evacuation of intracranial mass lesion was done in 14%, and decompressive craniectomy was performed in 2% (Table 1).

**Fig. 2:**
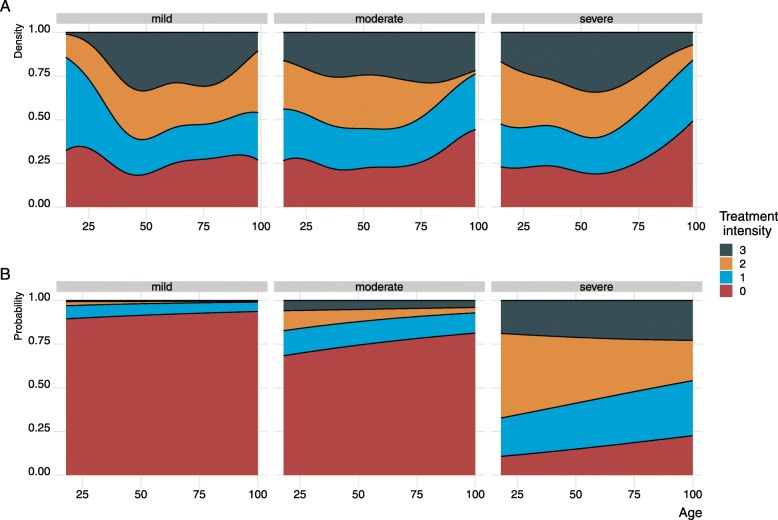
The effect of age on management intensity (= composite score) of admitted TBI patients. **a** The resulting density of management intensity versus age as a continuous variable. **b** The modelled probabilities of the different treatment intensities. Both graphs demonstrate that increasing age is associated with less management intensity irrespective of head injury severity

Trauma team activation, advanced TBI imaging, invasive ICP monitoring, and ventilator treatment all declined significantly with increasing age (*p* < 0.001). There was no significant change in the rate of surgery with increasing age for mild and moderate TBI, but for severe TBI the age curve was parabola shaped, indicating a low rate of surgery in both young and elderly patients compared to the middle-aged patient group. Decompressive craniectomy was not performed in any patients ≥ 75 years. Figure 2 shows the effect of age on the management intensity (composite score). The graphs demonstrate that increasing age is associated with lower management intensity irrespective of head injury severity.

### Mortality

**Fig. 3:**
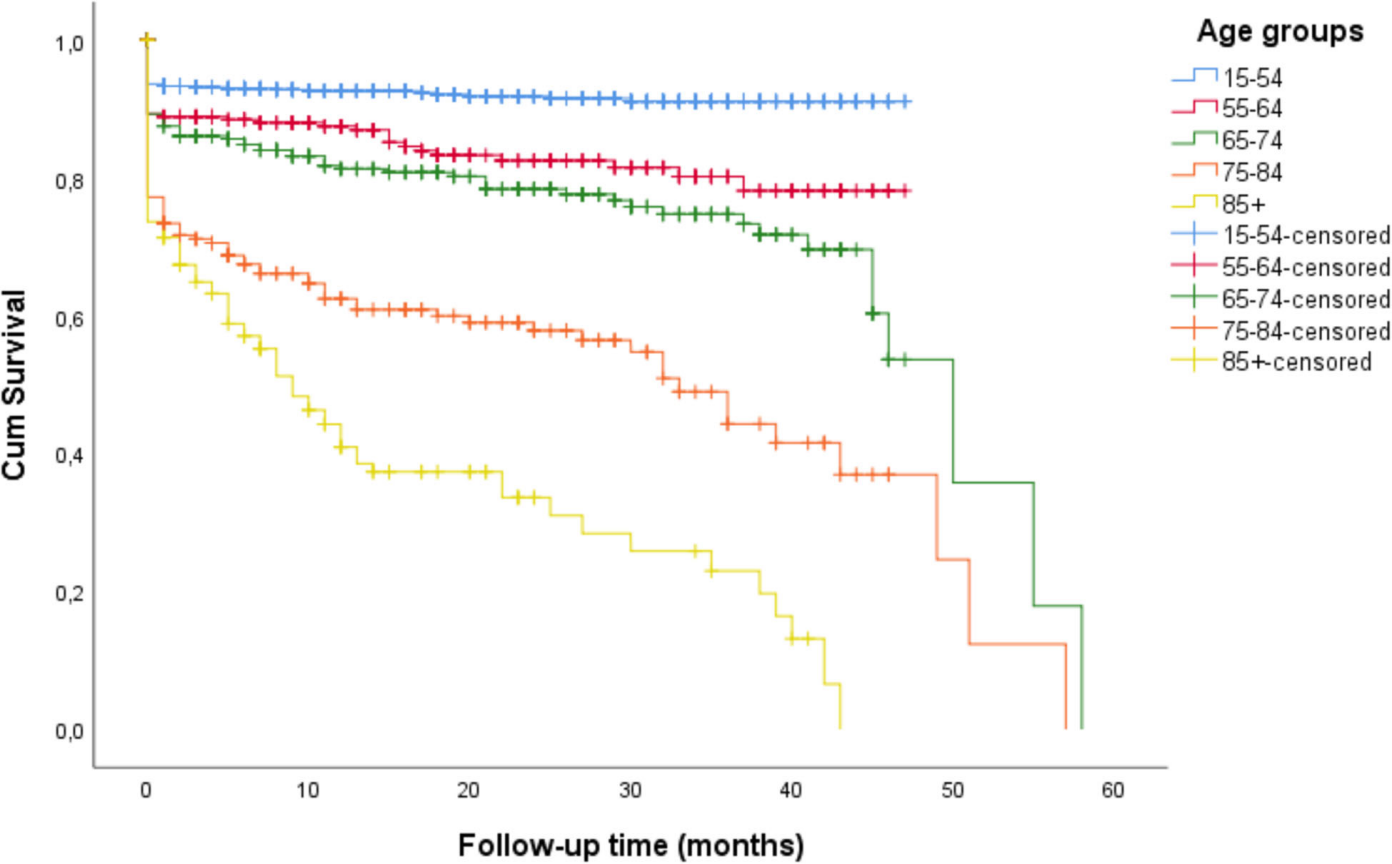
Kaplan Meyer plot of overall survival after TBI. The plot is showing poorer survival rate with increasing age of the patient

Overall survival analyses were conducted using Kaplan–Meier curves (Fig. 3). 30-day overall mortality was 12%. 30-day mortality in the age groups 15–54 years, 55–64 years, 65–74 years, 75–84 years and ≥ 85 years was 6%, 11%, 11%, 23% and 24%, respectively. Uni- and multivariate survival analyses were performed using logistic multinomial regression (Table 2). In the multivariate analyses, the following parameters had a significant association with an increased risk of death within 30 days of trauma: increasing age, severe comorbidities, severe TBI, Rotterdam CT-score ≥ 4, and low management intensity. Sex was not associated with 30-day survival.

**Table 2:** Cox regression analyses of parameters potentially associated with risk of death within 30-days of trauma

	**Univariate**			**Multivariate**		
	**OR**	**95% CI**	***p-value***	**OR**	**95% CI**	***p-value***
**Age**	1.03	1.02 to 1.04	< 0.001	1.00	1.00 to 1.00	< 0.001
**Sex**						
Female	—	—		—	—	
Male	0.85	0.62 to 1.19	0.35	1.02	0.99 to 1.05	0.20
**Preinjury ASA**						
Healthy	—	—		—	—	
Moderate disease	1.58	1.02 to 2.43	0.038	0.99	0.96 to 1.03	0.75
Severe disease	3.57	2.45 to 5.27	< 0.001	1.06	1.02 to 1.10	0.004
Life-threatening	3.82	0.84 to 12.8	0.045	1.09	0.95 to 1.25	0.20
**Management intensity**						
0	—	—		—	—	
1	8.82	5.93 to 13.3	< 0.001	1.06	1.02 to 1.10	0.005
2	2.79	1.71 to 4.50	< 0.001	0.91	0.87 to 0.95	< 0.001
3	4.60	2.74 to 7.62	< 0.001	0.82	0.77 to 0.87	< 0.001
**TBI severity**						
Mild	—	—		—	—	
Moderate	3.77	1.57 to 11.2	0.007	1.03	0.99 to 1.06	0.11
Severe	30.4	13.7 to 86.4	< 0.001	1.23	1.17 to 1.28	< 0.001
**Rotterdam CT score**						
1,2	—	—		—	—	
3	2.00	1.17 to 3.53	0.013	1.00	0.97 to 1.03	0.85
4	9.16	5.05 to 17.1	< 0.001	1.19	1.13 to 1.26	< 0.001
5	28.0	15.5 to 52.5	< 0.001	1.48	1.39 to 1.57	< 0.001
6	111	50.1 to 268	< 0.001	1.86	1.71 to 2.02	< 0.001

*OR* Odds ratio, *CI* Confidence interval, *ASA* American Society of Anesthesiologists

## Discussion

The present study indicates that the management intensity of hospitalised patients with TBI decreased with advanced age and that low management intensity was associated with an increased risk of 30-day mortality. Thus, we cannot rule out that the higher mortality among elderly TBI-patients has an element of self-fulfilling prophecy.

### Age and sex

Seventy per cent of our patients were men. The male preponderance was very clear in younger patients, while in older patients this difference was less pronounced. In patients ≥ 85 years there was a female preponderance. The total number of patients with TBI aged ≥ 55 years exceeded the number of patients < 55 years. In sum, there are still many young males admitted for TBI, but they are outnumbered by men and women ≥ 55 years. The age and sex distributions found in this study are in line with several recently published studies [3, 6, 31].

### Age and comorbidities

In this study, increasing age was significantly associated with severe systemic disease, need for assistance in daily life, and the use of antithrombotic medication. The close association between older age and comorbidity is in line with other recent TBI studies [3, 32, 33]. Antithrombotics are associated with increased risk of intracranial hematoma after blunt head injury, progression of intracranial hematoma, increased morbidity, and mortality in the TBI population [34–37]. Based on observation in clinical practice, comorbidities appear to be a more important factor than age itself for treatment decisions in patients with TBI.

### Age and injury mechanism

Falls were the most frequent injury mechanism, and the proportion of fall-related injuries increased gradually with increasing age, which is in line with other recent TBI studies [3]. Thus, the typical trauma patient today is a man or woman ≥ 50 years old with a low-energy fall injury. The World Health Organisation has defined risk factors for falls, and these include polypharmacy, comorbidities, age > 80 years, impaired cognition (especially attention and executive dysfunction), impaired vision, and environmental factors [38, 39].

### Age and injury severity

The severity of TBI, as assessed by GCS and HISS, tended to decrease with increasing age, whereas TBI severity assessed by the Rotterdam CT score showed a gradual increase in severity with increasing age. This discrepancy is somewhat surprising but may perhaps represent known limitations of GCS and HISS (i.e., they are poor discriminators of less severe TBI). However, an increased fraction of less severe TBI in older adults has been reported and been linked to more frequent low-energy traumas in this age group [3]. This link is supported by the lower number of multiple traumas that we found among the older adults in our study [3].

### Age and management intensity

As markers for management intensity in the different age groups, we used rates of trauma team activation, advanced TBI imaging, invasive ICP-monitoring, ventilator treatment, surgical evacuation of mass lesion, and decompressive craniectomy. All six parameters showed a declining rate of administration with increasing patient age. Invasive ICP-monitoring, ventilator treatment, and surgical evacuation of mass lesion are directly treatment related, and were included in the composite score of management intensity. The composite score, as visualised in Fig. 2, demonstrates that increasing age was associated with reduced management intensity irrespective of head injury severity. Decompressive craniectomy was not included in the composite score, because it must still be regarded as a treatment with limited documented benefit and is a treatment hardly documented at all in patients ≥ 65 years [40, 41].

Invasive ICP-monitoring of patients with TBI, according to the recommendations by the Brain Trauma Foundation, has been proven beneficial [42]. The reduced use of ICP-monitoring with increasing age has been reported before [10, 42], and a low rate of surgical evacuation of traumatic intracranial mass lesions in TBI patients ≥ 65 years is in line with previous reports [10, 43]. Bus et al. suggested that the tendency to restrict surgical treatment in the elderly is because of presumed poor prognosis and may have acted as a self-fulfilling prophecy [43]. Whitmore and colleagues wrote in 2012: “*When all the costs of severe TBI are considered, aggressive treatment is a cost-effective option, even for older patients. Comfort care for severe TBI is associated with poor outcomes and high costs, and should be reserved for situations in which aggressive approaches have failed or testing suggests such treatment is futile*” [21]. Kirkman et al. published a study in 2013 on TBI in the elderly and presented national data from UK hospitals showing that time from admission to CT head imaging increased with increasing age, as did the likelihood of not being transferred to a centre with acute neurosurgical care facilities and being reviewed only by the most junior grade doctor.

### Age and mortality

The 30-day mortality in this cohort of hospital-admitted patients with TBI identified by neuroimaging was 12%. In multivariate analyses, increasing age, severe comorbidities, increasing severity of head injury, and low management intensity were significantly associated with increased risk of 30-day mortality. These predictors of TBI mortality are in line with other TBI studies [30, 44–48]. The associations between age, management intensity and mortality are intriguing and should be assessed in more detail in future studies, especially because the severity of TBIs tends to be lower in elderly patients than younger ones.

### Self-fulfilling prophecy?

The two main findings in this study are the reduced management intensity with increasing age and the association between management intensity and risk of 30-day mortality. Whether this reduced management intensity in elderly patients represents well-considered treatment-limiting decisions in selected patients or suboptimal treatment remains unanswered. Thus, we cannot rule out the possibility that the high mortality and morbidity among elderly TBI patients might partly be explained by a self-fulfilling prophecy. The answer to this somewhat provocative question may probably be found in large multicentre comparative effectiveness studies like the CENTER-TBI in Europe and TRACK-TBI in the US [49, 50]. There is also a need for more qualitative research addressing decision-making rules for treatment-limiting decisions in TBI patients among physicians, nurses, and other health care providers [51]. During the last 30 years there has been a significant change in attitude to treating older patients for severe medical conditions, e.g. in cardiology, cancer, and degenerative spine conditions [52–55]. Increased knowledge and improvements during the last decades in anaesthesiology, intensive care medicine, neurosurgery, advanced surgical techniques, and rehabilitation give us the opportunity to push the previous limits of TBI treatment [56–59].

### Strengths and limitations

The present study includes hospital-admitted patients with acute TBI identified by neuroimaging. If patients are triaged after recommended guidelines, the vast majority of adult patients with TBI admitted to the hospital will have intracranial injuries identified by neuroimaging [25]. Thus, the patients included in this study will most likely be representative of the majority of Level 1 trauma hospital-admitted patients with TBI.

A substantial number of trauma patients are first triaged at local hospitals in our health region. The referral practice of these patients to the level 1 trauma centre may depend on the age and comorbidities of the patients. Therefore, we have reason to believe that many older adults with comorbidities and severe injuries are never referred. If this is correct, the management intensity of patients with TBI in the upper age groups is even lower than reported in this study.

We present no direct proof that the high mortality among elderly TBI patients can partly be explained by a self-fulfilling prophecy. Nevertheless, we still believe it is appropriate at least to consider this possibility in light of recent data indicating the benefit of aggressive acute treatment and rehabilitation in older patients with TBI [18–23].

## Conclusion

The present study indicates that the management intensity of hospitalised patients with TBI decreased with advanced age and that low management intensity is associated with an increased risk of 30-day mortality. Thus, we cannot rule out that the high mortality among elderly TBI patients could have been limited with a more aggressive management regime.

## Data Availability

The datasets generated and/or analyzed during the current study are not publicly available due to the sensitivity of the material, but are available from the corresponding author on reasonable request.

## Abbreviations

ASA: American Society of Anaesthesiologists
CT: Computed Tomography
CTA: Computed Tomography Angiography
CTV: Computed Tomography Venography
DPO: Data Protection Officer
GCS: Glasgow Coma Scale
HISS: Head Injury Severity Scale
ICP: Intracranial Pressure Monitoring
MRI: Magnetic Resonance Imaging
MRA: Magnetic Resonance Angiography
MRV: Magnetic Resonance Venography
OS: Overall Survival
OUH: Oslo University Hospital
REC: Regional Ethical Committee
TBI: Traumatic Brain Injury
TTA: Trauma Team Activation
TLD: Treatment Limiting Decisions
